# Using Sensor Data to Detect Lameness and Mastitis Treatment Events in Dairy Cows: A Comparison of Classification Models

**DOI:** 10.3390/s20143863

**Published:** 2020-07-10

**Authors:** Christian Post, Christian Rietz, Wolfgang Büscher, Ute Müller

**Affiliations:** 1 Physiology Unit, Institute of Animal Science, University of Bonn, 53115 Bonn, Germany; ute-mueller@uni-bonn.de; 2 Department of Educational Science, Faculty of Educational and Social Sciences, University of Education Heidelberg, 69120 Heidelberg, Germany; christian.rietz@ph-heidelberg.de; 3 Livestock Technology Section, Institute for Agricultural Engineering, University of Bonn, 53115 Bonn, Germany; buescher@uni-bonn.de

**Keywords:** classification, sensor data, lameness, mastitis, machine learning

## Abstract

The aim of this study was to develop classification models for mastitis and lameness treatments in Holstein dairy cows as the target variables based on continuous data from herd management software with modern machine learning methods. Data was collected over a period of 40 months from a total of 167 different cows with daily individual sensor information containing milking parameters, pedometer activity, feed and water intake, and body weight (in the form of differently aggregated data) as well as the entered treatment data. To identify the most important predictors for mastitis and lameness treatments, respectively, Random Forest feature importance, Pearson’s correlation and sequential forward feature selection were applied. With the selected predictors, various machine learning models such as Logistic Regression (LR), Support Vector Machine (SVM), K-nearest neighbors (KNN), Gaussian Naïve Bayes (GNB), Extra Trees Classifier (ET) and different ensemble methods such as Random Forest (RF) were trained. Their performance was compared using the receiver operator characteristic (ROC) area-under-curve (AUC), as well as sensitivity, block sensitivity and specificity. In addition, sampling methods were compared: Over- and undersampling as compensation for the expected unbalanced training data had a high impact on the ratio of sensitivity and specificity in the classification of the test data, but with regard to AUC, random oversampling and SMOTE (Synthetic Minority Over-sampling) even showed significantly lower values than with non-sampled data. The best model, ET, obtained a mean AUC of 0.79 for mastitis and 0.71 for lameness, respectively, based on testing data from practical conditions and is recommended by us for this type of data, but GNB, LR and RF were only marginally worse, and random oversampling and SMOTE even showed significantly lower values than without sampling. We recommend the use of these models as a benchmark for similar self-learning classification tasks. The classification models presented here retain their interpretability with the ability to present feature importances to the farmer in contrast to the “black box” models of Deep Learning methods.

## 1. Introduction

Supporting herd managers to identify animals with health problems is an important task of precision livestock farming. The automation of dairy farms as well as the size of dairy herds is continuously increasing [1] which makes it necessary to support farmers with digital decision support systems, and ensure the welfare of the cows not only for economic reasons, but also against the background of animal protection laws and ethics. A large number of studies already exist that have developed and evaluated models for classifying cows in need of treatment for mastitis [2,3,4,5] and lameness [6,7,8,9] with different machine learning methods, such as logistic regression [8,10], support vector machines [6], Bayesian classifiers [3,4] and neural networks [2,11]. These studies are usually limited to testing a single model with different conditions, so they can only be compared to a limited extent. These studies use different independent variables as features, and the machine learning models used differ in their ability to represent the importance of the features in relation to the target [12].

Furthermore, the reference method for defining the positive case varies between systematic veterinary examinations of all animals [13], assessment of the gait of cows [6,10,14] and milk [14], respectively, and records of mastitis or lameness treatments [3,7]. A lack of a standard for the target characteristic makes it impossible to compare different publications, especially in the case of lameness detection, as the visual assessment of lameness is to some extent subjective, and there are more than 20 different scoring scales [15]. In addition, cows and days were sometimes selectively included in the test data [13] or unexplained cases were removed from the test data [8,16]. These circumstances also change the frequency of occurrence of the target variable, i.e., the probability of whether an animal has undergone treatment or not, in the data. An artificially high frequency allows for higher combinations of sensitivity and specificity than what would be the case in practice [15].

The aim of the present study was to apply a variety of machine learning models, e.g., logistic regression, support vector machines and decision tree-based models, and different methods of sampling (random under- and oversampling, SMOTE) to a practical data set in order to identify the most important features and make daily classifications of cows for mastitis and lameness treatments. The results are compared using the receiver operator characteristic (ROC) as well as the combination of sensitivity and specificity, and will finally be used to make recommendations for further experiments of this kind.

## 2. Materials and Methods

### 2.1. Data Source and Preprocessing

Raw data was collected from the dairy herd at Frankenforst research farm of the University of Bonn. The herd on average consists of 65 German Holstein dairy cows with 305 day milk yield of 9605 kg. All data from the farm was collected, processed via an SQL database system and presented as CSV files. The raw data comprised a period from June 2015 to October 2018 and contained in total 167 different animals. The data set consisted of individual animal information (animal ID, parity, days in milk) with one record per cow per day (*n* = 80,307). Milking data (milk yield, duration, milk flow, and conductivity) was recorded twice a day. Feeding (roughage and concentrate from automated feeders) and drinking data contained the amount per visit, number and time of visits as well as visits without intake. Activity was measured as the sum of impulses from a pedometer at a 2 h resolution, and climate data (temperature, humidity) from a nearby weather station was present as daily aggregations. The data also contained monthly milk recordings (fat, protein and lactose content, and somatic cell count in 1000 cells/mL), which was copied to each day for each cow until the next successive recording, so that each day contained the information about the data from the last milk recording. Lastly, all recordings of veterinary treatments, hoof trimmings and other routine measures were added to the data, each with a category and diagnosis. Cows in need of a treatment were identified by farm staff during the work routine, and treatments were conducted by a trained veterinarian.

Before further processing, the data was checked for plausibility: for feed intake, water intake, and visit duration all values that were more than 3 standard deviations above or below the herd mean were removed, as well as absolute values below 5 kg and above 100 kg for feed and 200 kg for water intake. Values above 15 kg for daily concentrate intake were also discarded. Values deleted in this process, as well as missing values in the raw data, were linear interpolated up to 7 d. Recordings with remaining missing values were discarded after this process.

#### 2.1.1. Additional Aggregation

The Pedometer activity and the feeding visit data were further aggregated with calculations presented in [8] (see Table 1 for variable descriptions). In addition, for each variable except parity, days in milk and weeks in milk, several aggregations over multiple consecutive days were calculated to capture their development over time for each cow, which are also described in Table 1.

From the data, all days with recorded lameness and mastitis treatments were extracted as the dependent variables (targets). For each lactation with at least one treatment, only the first treatment was considered and the remaining days were discarded. Because of a high probability that the sensor data on the treatment day was influenced by the treatment itself, the day of treatment was then shifted back one day. In addition, all 3 days prior to a treatment were also considered as a positive target to calculate block sensitivity (for a definition see Section 2.5), as done in [7,17].

#### 2.1.2. Data Splitting

For each classification of lameness and mastitis cases the data set was split 10 times into training and testing data for model evaluation. Sixty six percent of individual cows were randomly sampled and all data points from those cows formed the training set. Consequentially, the test set contained the remaining data. In both data sets, days in lactations that contained a lameness or mastitis treatment were discarded after the first treatment occurred. For lactations without a treatment, a random day was chosen for cut-off instead. The data in the training set was further reduced to four weeks per lactation. Finally, the data in both sets was scaled by subtracting each feature’s mean and dividing by its standard deviation (Z-score normalization), because some models, like KNN and SVM, assume that all data is within a similar range.

### 2.2. Feature Selection

To lower the training time and overfitting of some algorithms (see discussion section), the number of independent variables (features) in the data was reduced. First an estimator (in this case Random Forest) was fit to the training data to obtain feature importances (RF-I) and biserial correlation (r), i.e., the average decrease in impurity for a feature over all trees [18]. The features were then sorted by their RF-I and the best 100 were kept. Further reduction was done by using Sequential Forward Selection (SFS) [19]. Here, the estimator is initialized with an empty feature subset, and in each iteration, one additional feature is added to the subset, performance of the estimator is measured and the feature associated with the best performance is removed from the feature set and added to the subset, until it reached a size of 20 features. The measure of performance was Matthew’s correlation coefficient since it is a performance metric suitable for imbalanced data [20]. This analysis was performed for both target variables (lameness and mastitis treatments).

### 2.3. Sampling Methods

The known low occurrence of treatments in the data results in a high imbalance between days with and without treatment. A common method of addressing this is to equalize the distributions of both classes by sampling. Three commonly used methods from the Python module *imblearn* [21] were applied to the training data: (1) Random Oversampling populates the data set with copies of randomly selected data points of the minority class (days with a treatment). (2) In Random Undersampling data points from the majority class (days without treatment) are removed at random. (3) SMOTE (Synthetic Minority Over-sampling Technique) is a variant of oversampling, where instead of copying existing data points, new (synthetic) data points of the minority class are created by creating a random vector between a data point and its k neighbors (k = 3 in this case) in the multidimensional feature space [22]. Each of these three methods resulted in a data set with an equal number of days with and without a treatment. The sampled training data sets as well as the non-sampled data were then used to train the classification models.

### 2.4. Classification Models

Data processing, model building and presentation of results was done with the Python language (Python Software Foundation, Wilmington, DE, USA). The following classification models that were used were all part of the module Scikit-learn [23]: Logistic Regression (LR), Support Vector Machine (SVM), K-nearest neighbors (KNN), Gaussian Naïve Bayes (GNB), Decision Tree Classifier (DT), Random Forest (RF), Extremely randomized trees, or ExtraTrees (ET), and AdaBoost (ADA). These represent a wide range of commonly used machine learning techniques.

LR calculates the probability of a data point to belong to one of two classes, in this case a day with a treatment. This is done by estimating the model parameters via maximum likelihood estimation. Given the probability, a threshold is then used to classify the data point [18]. To compensate for possible multicollinearity in the feature set and improve coefficient estimates, the l2 penalty (ridge regression) was added to the model. A SVM constructs a linear decision surface, also known as hyperplane, in a multi-dimensional feature space that has the widest possible margin to separate data points of both classes. To achieve non-linear separation, the input vectors are mapped to a higher dimensional space with a kernel function [24]. In KNN classification, a data point is assigned to one class by a majority vote of its k neighbors. The metric used to identify the neighbors is the Euclidean distance in the feature space [25]. In this study the default value of 5 was used for the number of neighbors. GNB estimates the probability of a cow having a treatment or not given the corresponding feature vector [18]. Though GNB makes the *naïve* assumptions that the input features are normally distributed and independent, the obtained binary classifications work reasonable in practice, even with violated assumptions [26]. A DT classifies data points by asking questions about the feature vector (called interior nodes) that eventually lead to one of many labelled end points (called leaf nodes). At each node, the feature to split the data is determined by a measure of purity of the daughter nodes, typically the gini impurity or the information gain [27].

#### Ensemble Methods

A model ensemble is a classification model that, instead of constructing a single model to make a prediction, generates a set of different models and combines their predictions into a single estimation. This is done by bagging, where each model of the ensemble is trained independently on a random bootstrapped sample of the training data, and boosting, where a strong classifier is built from a set of multiple weak classifiers [28]. In a RF, decision trees are built with only a limited, randomly drawn selection of features at each node [29]. In contrast to decision trees, the random sampling of each tree makes the random forest less susceptible to over-fitting [18,29]. An important hyperparameter for this algorithm is the number of features selected at each node, which here was set to √F, where F is the number of total features in the data set [18]. Furthermore the number of trees was set to 500 and the maximum depth was not limited. ET is a derivation of the random forest algorithm, where in addition to picking features at random for each split, the threshold that splits the data is also drawn from a randomly generated set of splits. This is done to reduce the variance even more in comparison to the random forest [23]. ADA creates an ensemble of weak classifiers that are restricted in their depth (also called stumps). These learners are used to give predictions on a modified set of the data, where each data point is given a weight. These weights decrease and increase for correctly and incorrectly classified data, respectively, meaning that those data points that are difficult to classify gain influence with each iteration. The classification of test data is done through a majority vote [18].

A Voting Classifier simply is an ensemble of arbitrarily selected classifiers. The decision on labelling a sample can either be done through hard voting, where the assigned class label is the majority vote of all classifiers, or soft voting, which takes into account the uncertainty of each classifier by using the weighted average class probability [23]. In this experiment, two different configurations were used: SoftVoting1 (RF, KNN, and GNB) and SoftVoting2 (LR, ADA and KNN).

### 2.5. Evaluation

For each classifier, a prediction of the probability of belonging to the class label 1, i.e., in need of a treatment, was given for each data point in the test data. The resulting vector of probabilities compared with the vector of true labels was used to create a Receiver Operator Characteristic (ROC) curve, which plots the rate of true positives over the false positive rate (1—specificity) for all different thresholds (thresholds that result in redundant combinations are excluded) [23]. This curve allowed to calculate the Area Under Curve (AUC), which is a value between 0 and 1, where 0.5 describes a random classification, and 1 would be a perfect match between classification and the target variable.

Before classification of the testing data, a subset of 33% of the training data was selected at random and used as a validation data set for which a classification was made. From the resulting ROC, the threshold was selected where the sensitivity was at least 0.8, and this threshold was used for classification of the testing data set.

The resulting true positives, false positives, true negatives and false negative were entered into a confusion matrix to calculate the specificity, as well as the positive and negative predictive values. In addition, the block sensitivity was calculated, where a true positive was at least one correct classification of the three days before a treatment, and a false negative if neither of these days was classified as a treatment.

All results are presented as the mean ± the 95% confidence interval of the mean. The measures of performance (AUC, sensitivity, block sensitivity and specificity) for classification models per sampling method (no sampling, Random Over- and Undersamling, SMOTE) were compared using the Welch’s test due to violated homogeneity of variance. These tests were implemented with SPSS version 26.0 (IBM Corp, Armonk, NY, USA) with significant differences at *p* ≤ 0.05.

## 3. Results

After processing and plausibility checks, 53,970 records remained in the data from 112 individual cows and 235 cows individual lactations, respectively. This corresponds to approx. 67% of the original data. Data from 55 cows was removed due to short lactations <10 d or incomplete sensor data.

### 3.1. Feature Importance

The process of variable creation described in Section 2.2 resulted in a total of 471 different independent variables (features). The total number of variables by category can be seen in Table 2. The most features were generated by sensors where data was available in a higher than daily resolution (feed and water troughs, pedometer activity). The milking variables included daily milkings as well as the last monthly milk recording. From all features, 83 represented data of a single day, while the other 350 described temporal relationships.

Feature importances (RF-I) and correlations (r) with the target variable (mean + 95%-CI) of the 20 most important features for mastitis treatments are shown in Table 3. 

#### 3.1.1. Mastitis Treatments 

The highest RF-I (0.039) as well as the highest correlation with the mastitis treatment events (0.176) was shown by the somatic cell count from the last monthly milk recording. It is remarkable that of the further variables listed, only two others can be associated with the daily milking data: The slope and the difference from the rolling mean of the evening milk conductivity (RF-I = 0.013, r = −0.076 and RF-I = 0.010, r = 0.080, respectively). The other 19 most important features consisted of temporal derived variables from the feeding troughs and the concentrate feeder. The slope of the absolute concentrate intake and the deviation from the allowance were both negatively correlated with the treatment. The feed intake variables showed positive correlations while the correlations of the feeding visits were negative.

#### 3.1.2. Lameness Treatments

RF-I for lameness classification were lower overall than for mastitis. Total time at the feeding trough with intake was most important (RF-I = 0.013) and showed a relatively high negative correlation (r = −0.105). The number of feeding and drinking visits and the time spent at the through also had negative correlation values. The four present activity features showed the standard deviation and range of daily values and had a negative correlation. Two climate features (temperature and THI) had a RF-I of 0.009 and a correlation of −0.061. The proportion of temporal derived features among the most important was lower than with mastitis.

### 3.2. Classification Results

#### 3.2.1. Results for Training Data

For a part of 33% of the training data (=validation data, sampled and without sampling) classifications were made by each machine learning method to obtain a limit value for the classification of the test data based on the resulting combinations of sensitivity and specificity. From these results, AUC as well as sensitivity and specificity could be calculated, providing an overview of the application of the methods to sampled data. These data are presented in Table 4 (mean values ± 95%-CI for each sampling method, results for all machine learning methods combined). From this, it can be seen that for both mastitis and lameness treatments, random oversampling and SMOTE are higher than random under sampling (0.76 and 0.71) and without sampling (0.80 and 0.76) for AUC, with 0.95 and 0.91 respectively.

#### 3.2.2. Results for Testing Data

**Figure 1:**
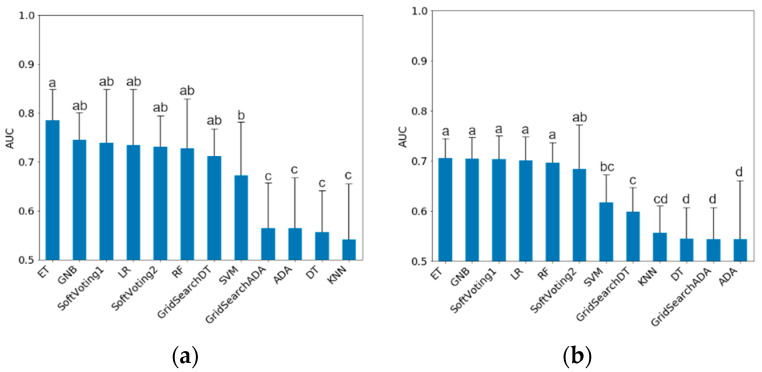
Mean test data AUC (Mean ± CI) for models trained on non-sampled data. (**a**) Mastitis treatments; (**b**) Lameness treatments. Different letters indicate significant (*p* < 0.05) differences between classification models. AUC: Area Under ROC-Curve; ET: ExtraTrees Classifier; GNB: Gaussian Naïve Bayes; LR: Logistic Regression; RF: Random Forest; SVM: Support Vector Machine; ADA: AdaBoost; DT: Decision Tree; KNN: K-Nearest Neighbors.

All following results refer to the classification of the test data. The mean AUC results of the classification models trained on non-sampled data are shown in Figure 1. For mastitis, ET, GNB, GridSearchDT, LR, RF and the Soft Voting ensembles showed the highest mean AUC with ET at 0.79 (0.73–0.84). Grid search improved the Decision Tree model, but not AdaBoost. There were no differences between the two Soft Voting ensembles (AUC 0.74 vs. 0.73 for mastitis and 0.69 vs. 0.66 for lameness, respectively). The overall variance in the mastitis classifications was higher than in lameness, as indicated by the larger CI.

**Figure 2:**
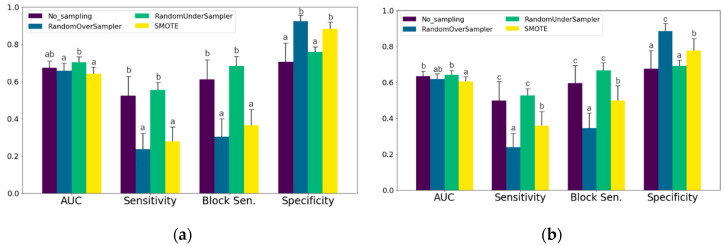
Mean test data AUC, Sensitivity, Block Sensitivity and Specificity (± 95%-CI) for each sampling method. (**a**) Mastitis treatments; (**b**) Lameness treatments. Different letters indicate significant (*p* < 0.05) differences between sampling methods. AUC: Area Under ROC-Curve; SMOTE: Synthetic Minority Over-sampling Technique.

To compare the sampling methods, Figure 2 shows the differences for AUC, sensitivity, block sensitivity and specificity between the sampling methods. For mastitis, Random Undersampling resulted in a higher AUC than both oversampling methods. For lameness the difference between Random Undersampling and Oversampling was not significant, but there was still a difference to the models that used SMOTE. Without sampling and with Random Undersampling the ratio of sensitivity to specificity is moving in favor of sensitivity for both mastitis Figure 2a and lameness Figure 2b treatments.

In order to test the effects of including the data from feed and water troughs, the complete evaluation was carried out both including these features and without. Table 5 shows the results for AUC, sensitivity and specificity, averaged over all sampling methods and machine learning models (mean values ± 95%-CI). It becomes clear that similar AUCs (0.67 and 0.66) were achieved in the classification of mastitis treatments both with and without the feed and water data, while the classification of lameness treatments suffered from the exclusion of these features (AUC of 0.62 vs. 0.55).

To finally compare the machine learning models in their ability to correctly classify mastitis and lameness treatments, the following Table 6 ranks them by AUC (mean ± 95%-CI) in descending order. The highest mean AUCs for the classification of mastitis treatments in the test data were obtained from LR, ET, GNB, Soft Voting 1 and 2, RF, and Grid Search DT with values between 0.75 and 0.69. For lameness treatments GNB, Soft Voting 1, ET, LR, RF and Soft Voting 2 yielded the highest AUCs between 0.70 and 0.66. In both treatment categories KNN, Grid Search ADA, ADA and DT resulted in the lowest AUC values between 0.58 and 0.54.

## 4. Discussion

### 4.1. Feature Importance

Feature importance obtained from Random Forest (RF-I) is commonly used to reduce the dimensionality of the model input. It is also interpreted to understand connections between the input and the output data, however, there are certain caveats such as that there is a bias against variables with only few categories [30] as well as a bias when variables are highly correlated [31]. This is why Sequential Forward Selection (SFS) was used as an iterative approach to reduce the feature set to its final size for model training and classification. Here, because only the feature that increases the applied measure (here: Matthews Correlation Coefficient, MCC) the most, non-correlated variables are favored. MCC was chosen because it takes into account all parts of the contingency table and thus is suited and recommended for imbalanced classification problems [20]. The advantage of this feature selection method is that it retains interpretability of the models for the farmer, which is not possible when using other methods such as Principal Component Analysis and Deep Learning, where the feature importance is harder to interpret. In practical applications, models that show the correlations between an alarm and the input variables could offer additional help for the farmer. Random Forest feature selection is suitable for reducing the number of input variables to the most important ones while retaining the interpretability of the final models.

For mastitis treatments classification, the SCC of the last monthly milk recording highlighted as the most important predictor with an RF-I of 0.039 and r = 0.176, despite the fact that time gaps between the last measurement and treatments could be as large as a month. An elevated SCC is a direct indicator for both subclinical and clinical mastitis, which also explains the comparably high positive correlation with the treatments. Former studies also showed that including SCC as a feature improved classification: On-line measured SCC improved the positive predictive value for clinical mastitis from 0.11 to 0.32 compared to electrical conductivity alone [32]. The incorporation of cow information that included previous SCC from milk recording also improved AUC of mastitis alerts from 0.62 (only information from AMS) to 0.78 [3]. The knowledge of the last SCC could have influenced the decision to conduct a mastitis treatment, but there was no systematic accumulations of treatments in the week after milk recording results were received. The on-farm protocol is that for a cow to be treated there have to be signs of abnormal milk, detected by visual observation or with the California mastitis test. Compared to the importance of the other features (for mastitis as well as lameness treatments) the RF-I of monthly SCC is by far the highest. Because it can be assumed that SCC is a predictor with a direct relationship to udder health, it is questionable whether the features with RF-I < 0.014 should be viewed as having only an indirect relationship to the target variable. A raise in milk conductivity is also an indicator commonly used to detect mastitis, although the correlation to SCC is low (r = 0.48) [32] and the absolute value is dependent on the animal [33]. This explains the occurrence of two conductivity variables that capture the change over time in the 20 most important features for mastitis treatment classification. The somatic cell count from the monthly milk recordings is by far the most important predictor for the classification of udder treatments, as it is directly related to mastitis.

Other important features for mastitis treatments classification were derived from feeding data (concentrate and roughage intake, and feeding visits). Concentrate intake slope and deviation from allowance seemed to be important with RF-I of 0.014 and 0.010, respectively, but are dependent on the cow’s milk yield, since maximum daily allowance is automatically adjusted by milk kg and days in milk. The features for roughage intake included the current and previous rolling means and were positively correlated with treatments (RF-I = 0.008–0.011, r = 0.052–0.068). This might be the result of cows with a high milk yield (and thus a higher feed intake) being at an up to 1.44 times higher risk for mastitis compared to cows with low milk yield [34]. Feeding data are not highly correlated with mastitis treatments. Therefore they are less suitable as predictors for classifying udder treatments.

Features derived from pedometer activity and behavior are most commonly used as predictors for lameness, e.g., differences in average activity between days [7,10], and accelerometer data like number and duration of lying and standing events [35]. The mean number of step impulses of lame cows is supposed to be lower than for healthy cows [6]. This is expected and also shown in then own study: The activity features from the data all had a negative correlation with the lameness treatments, although this effect was small (r = −0.072). Pedometer activity has a significant, albeit small, correlation with lameness treatments.

In data from 118 cows (44 with a lameness-related treatment within a period of 10 months), activity variables (e.g., sum, mean and standard deviation of 12 daily 2 h-activity values, deviation from previous day, difference between previous weeks) showed a correlation of r = 0.23 ± 0.06 on average [8]. Here, neck collars were used that delivered activity indices based on number, intensity and direction of impulses. The resulting sensor data might have a more direct correlation to lameness than impulses measured by a leg pedometer. But [8] built their database by strictly excluding cows with treatments other than lameness, which also contributed to a higher correlation with lameness treatments because there was less noise in the data from other treatments. In the own study, the aggregated impulse data from leg pedometers was used, because this sensor is available on most practical farms nowadays. As expected, the results show that they cannot be considered a sensor with a direct correlation to lameness treatment, unlike the monthly SCC for mastitis. This can be explained with a high variance between individual lameness events and their impact on sensor variables [9]. Additionally, there are more different underlying conditions for lameness (e.g., sole ulcers, sole hemorrhages, or digital dermatitis), which have a different impact on a cow’s gait [36]. Other sensors like automatic gait score assessment with cameras, or leg weight distribution can potentially improve classification, as seen in [37] where lameness (based on scores) was detected from leg weight distributions on a weighing platform, with an AUC of up to 0.88. This value cannot be compared to the own study though, because the underlying data set consisted only of 7 d and a single classification, based on clinical examinations of all cows. These advanced sensor systems are not widely available in practice. This emphasizes the need for sensors that are more directly related to lameness treatments than just the number of activity impulses.

Data of roughage and water intake and visits is obtained from weighing troughs that are only used in experimental dairy farms, but not usable in practice. Information about a cow’s feeding and drinking visits can be approximated with the use of tracking [38] and accelerometer systems [39], but the amount of roughage and water intake of individual cows remains unknown. When excluding all features derived from the weighing trough data in the own study, a mean AUC of only 0.55 was obtained for lameness classification, confirming the importance of those features. Two features from water troughs, drinking time (RF-I = 0.009, r = −0.065) and rolling mean of the number of drinking visits (RF-I = 0.006, r = −0.059) were found among the 20 most important features. This indicates a potential for water trough visits as a feature for lameness classification. From a technical point of view, it is conceivable that in the future these characteristics could be recorded with the help of sensors. Data from feeding and water troughs improve the classification of lameness treatments, but are not available as such in practice.

### 4.2. Sampling Methods

The goal of over- and undersampling is to mitigate effects of severe class imbalance, as seen in the data used in the own study, where samples labeled as positive were less than 1% of the data set. The own results showed no improvement in AUC for testing data when using sampling methods on the training data. Few studies on the classification of treatments or health related events in dairy cows explicitly use over- or undersampling techniques. One study that tried to identify cows with a high locomotion score used SMOTE to balance data with 45 lame and 1.613 non-lame days to achieve a sensitivity of 1.00 (95%-CI: 0.19–1.00) and specificity of 0.8 (95%-CI: 0.71–0.87) on validation data (10% of total data), but they did not compare that to a model trained on non-sampled data [40]. When classifications were made for sampled data (validation data, see Section 2.5), SMOTE and Random Oversampling, compared to non-sampled data, both improved the AUC for mastitis (0.95 vs. 0.80) and lameness (0.91 vs. 0.76). In the study of [41] different intensities of random sampling were applied to a credit score classification problem. The portion of negative samples in the data ranged from 1% to 30 % and showed that some models (LR, linear SVM and quadratic linear discriminant analysis) suffer from high imbalances and resulted in AUC values of nearly 0.50 for data with 1% negative samples, while decision tree-based models were less affected (highest AUC for the 1% negative set of 0.90). However, in that study the testing data was also sampled. But for a robust estimation of a model’s capabilities in a practical setting, sampling cannot be applied to the testing data, because that would require a priori knowledge of the true underlying condition (a cow is needing a treatment), which is not present in an applied use case. To make estimations for sensitivity, specificity, and AUC, the class distributions in the testing set should reflect those of real world data [18]. Over- and undersampling is commonly used to correct the imbalanced ratio between days with and without treatment in the training data, but this is not advisable for the testing data. Therefore, in the testing data this ratio should correspond as closely as possible to data from practical farms.

### 4.3. Interpretation of the Final Classification Models

Due to differences in the study design and the composition of the data, a direct comparison of the own results of the classification models with those of other studies dealing with the classification of dairy cows in need of treatment is only possible to a certain extent, even if the statistical methods are the same [42]. 

Studies that have also developed models to classify lameness or mastitis treatments as target variables differ in the independent variables (features) used. Studies that have classified cows for lameness treatment have either focused on feeding related variables such as feed intake, trough time or number of visits [43], or only on ALT pedometer data such as number of impulses and resting time [6] with a resulting accuracy of 0.76, or used multiple data sources (live weight, pedometer activity, milk yield) and additional individual animal information [10] to obtain an AUC of 0.74 compared to models with only a single parameter that yielded AUCs of 0.60–0.66. The own models used sensor data that are widely available in practice (pedometer activity, milking parlor data, concentrate intake, live weight, climate data, and monthly milk recordings) as well as data from feeding and drinking troughs (visits and intake). The classification for mastitis treatments in previous studies was based on activity and rumination time [5] with a sensitivity of 0.55, or change in milk yield and conductivity [44] to obtain a sensitivity of 0.48 when specificity was set to 0.98. [4] included both data from real-time milk analyzers (SCC, fat, protein) and non-sensory information (parity, season, and weeks in milk) in their model, which resulted in an AUC of 0.89. In the own study mainly sensor data and information were used that are already available on many farms, and we recommend this approach when developing classification models for practical application. The feed and water intake is a special case, because the data acquisition is only possible on experimental farms with weighing troughs. Therefore the evaluations were carried out also without these features. The results show that for the classification of mastitis treatments, the exclusion all features from feed and water troughs did not affect the AUC, while for the lameness treatment classification the mean AUC dropped from 0.62 to 0.55. This emphasizes the importance of those features and shows the need to develop and improve sensors that measure feeding behavior of individual cows.

The data sets also differ between the studies in terms of the different pre-selection options. This selection takes place before the separation into training and test data and therefore affects the relationship between treated and non-treated cows, which leads to a higher probability of correctly positive classifications. In the study of [6] a total of 549 days out of 11 cows were selected, of which about half were classified as “lame” based on lameness scores. Other studies excluded unclear cases from their data: Exclusion of cows with a scoring system from 1 (non-lame) to 4 (severe lameness) [16] or 1 to 5 [35] and exclusion of all animals with a score of 2 in both cases, or data for cows with more than 50% missing sensor data for activity were excluded [35]. Another possibility is to limit the test data to a certain number of days before treatment, e.g., 3 weeks, resulting in a higher proportion of days with treatment [8]. In the studies mentioned above, it is questionable which values for sensitivity and specificity would have resulted from less selected data. In our own study, only the days after treatment were not considered in the test data, missing values were interpolated. Values for sensitivity and specificity of up to 0.79 for mastitis treatments and 0.71 for lameness treatments could be achieved on the non-sampled test data. Excluding data and thus artificially increasing the number of positive cases in the test data leads to higher values for AUC, sensitivity and specificity and is therefore not recommended. Test data sets should, as far as possible, be similar to the data that will need to be classified later in practical use on farms. Many studies pre-select both training and test data, which leads to higher AUCs, but these are not achieved in practice. In future studies, classification models from sensor data should be tested on practical data.

In addition to the above-mentioned methods of processing the test data set, it is possible to increase the number of days with treatment by also defining a certain number of days before a treatment is carried out as a “day with treatment”, i.e., as a positive target variable. In our own study, 3 days were used, as in [7] for mastitis and lameness treatments. This allows the calculation of block sensitivity, where at least one of the three days prior to treatment must have been positively classified. In [7] block sensitivities of 0.77 for mastitis and 0.74 for lameness treatments were achieved, but no sensitivities per day were given for comparison. The highest block sensitivities in our own experiment were 0.80 for mastitis treatments and 0.81 for lameness treatments. In a practical setting on a farm, the exact day before a necessary treatment is not important as long as the need for treatment is visible. A disadvantage is that this can lead to animals in need of treatment being detected too early without clinical signs and then incorrectly registered as healthy, as has been discussed in other literature [9,42]. The calculation of block sensitivity reflects the use of a model in practice better than pure sensitivity for the exact day before treatment and is therefore recommended for the evaluation of classification models for mastitis and lameness treatments.

Finally, the recording, or definition, of the target variable also influences the interpretation of the results. In our own study, mastitis and lameness treatments were carried out by veterinarians and stable personnel, respectively. Also in other studies on classification models of dairy cow data, treatments of mastitis [3,4,7,43,45] and of lameness [7,8,43] were used as target variables. Although these records are made by qualified professionals and are therefore considered reliable, it was discussed to what extent this type of data collection incorrectly records cows without clinical signs as not requiring treatment [15,46]. Other studies have used lameness scores or clinical assessment to define lameness [10,35,36] and cell count measurements or cytobacteriological tests for mastitis [5,14]. This type of recording potentially reflects the health status of the individual animals better, but is much more personnel and time intensive and is therefore not suitable for evaluations over periods of several years. In addition, the use and storage of treatment data collected under practice conditions allows the underlying classification models to continuously adapt and learn from newly entered data to improve the classification of future treatment events.

So far there are few studies that systematically compare the application of different machine learning models for the classification of farm animal data. A study by [47] compared the methods RF, SVM, KNN and ADA for the classification of three different types of grazing behavior in sheep. There, RF gave the best results (highest accuracy of 0.92), with KNN and ADA worse by 0.05 on average. The authors particularly emphasized RF’s ability to correctly assess non-linearly related data and its robustness against statistical noise. The authors of [48] compared different methods (NB, DT, RF, Bayesian network and bagging) to predict insemination success in Holstein dairy cows based on phenotypic and genotypic traits. Results for AUC ranged from 0.61 to 0.75, with RF showing significantly better results than all other methods tested. Two studies from other disciplines are also mentioned here, credit assessment [41] and Alzheimer diagnostics [12], as they also systematically compared classification models on a binary target variable. In the study by [41] data sets with different proportions of good and bad credit scores were created and classified by means of down-sampling. Decision tree-based models (RF and gradient boosting) provided the highest AUC (up to 0.90), while other models such as LR and SVM showed only random classifications (AUC = 0.5) for the data sets with the lowest proportion of bad scores. The Alzheimer study, which used features of electrical brain activity, compared RF, SVM, LR and neural networks and found only minimal differences in AUC (0.83–0.87). The authors emphasize the advantages of models that output feature importance (RF, LR) compared to (non-linear) SVM and neural networks, whose decision making is much more difficult to interpret [12] and are therefore considered “black boxes”. In our own study, models based on decision trees (ET, RF) or containing them (Soft Voting 1), but also LR and GNB resulted in the highest average AUC (0.71–0.79 for mastitis treatments and 0.67–0.71 for lameness treatments, respectively).

Random forest models are more robust compared to single decision trees, because the tendency to overfitting and the overall variance within the model is lower [18]. They also perform well when dealing with class imbalances [41]. Logistic Regression has also been used for classification of treatments in other studies [8,10,49] and has the key advantage of interpretability, where not only the absolute importance of each feature, but also the trend can be derived from its coefficients [18,23]. Based on the results of this study the use of ET, LR, RF and GNB can be recommended for further, similar studies.

An AUC of 0.75 or 0.70, as achieved by the best own models for detecting mastitis and lameness treatments with a practical data set, is within the range of studies that have worked with similar data and sensor combinations. A value of 1 would mean a perfect classification. A value above 0.70 is considered a “strong model”, while a value below 0.60 is considered a “weak model” [28]. The more directly the features are related to the target/classification variable, the greater the AUC, as can be seen from the example of somatic cell count in relation to mastitis treatments in the own data. Sensors and features with a direct relationship to the target variable are required to achieve a higher AUC. For the detection of dairy cows with classification models no minimum requirements for AUC values are known. Various authors demand minimum sensitivity values of 0.70 or 0.80 with a specificity of 0.99 [15], which would correspond to an AUC of more than 0.90, and cannot be achieved with practical data sets (i.e., not sampled or similar). Only a few studies critically question the practical applicability of models with lower than the required AUC (or sensitivity and specificity combinations), e.g., in a study on calving prediction, where the authors point out the model’s limited benefit resulting from a low frequency of occurrence of the target variable “calving” in the data, and the resulting low positive predictive value despite AUC values of up to 0.81 [50]. The authors’ own AUC values also make it clear that the application of classification models from practical sensor systems on realistic data sets must be viewed critically and does not reliably lead to the identification of animals in need of treatment or similar classification events, as generally expected. Thus, limits of the use of sensors (the corresponding machine learning methods and all other associated techniques) for finding individual animals in need become apparent.

## 5. Conclusions

The following recommendations for future sensor-based classification models for single animal-related events result from the comparison of different evaluation variants and models on a comprehensive test data set: (1) The mastitis classification resulted in an overall mean AUC higher by 0.05 than the lameness classification, due to predictors with a higher correlation to the treatment (especially somatic cell count from monthly milk recordings with r = +0.18). (2) Over-and under-sampling of days with treatments in the training data did not improve the AUC when classifying the testing data, where the class balance should not be artificially increased and always reflect practical data. (3) The use of treatments as the target variable is required when using practical data over long periods of time and enables the use of potential self-learning models, which would not be possible with clinical observation. (4) The classification models presented here retain their interpretability with the ability to present feature importance and their significance to the farmer. This is an advantage over “black box” models such as deep neural networks, and should be considered in future models. (5) The best models, LR, ET, RF, GNB, and Soft Voting 1 and 2, obtained a mean AUC of 0.72–0.79 for mastitis and 0.66–0.71 for lameness, respectively, based on testing data from practical conditions and are recommended by us for this type of data.

This study shows that the classification of treatments using practical sensor data achieved similar results as comparable studies, with more classical methods (logistic regression) performing as well as newer methods (ExtraTrees, Gaussian Naïve Bayes). In a follow-up study, the transferability of the sensitivities and specificities obtained to practical data from dairy farms should be investigated and discussed.

## Figures and Tables

**Table 1:** Per variable description of additional aggregation of sensor data, daily (feed and water intake data, and pedometer activity) and over multiple consecutive days (all variables except parity, days in milk and weeks in milk).

Aggregation	Description
Daily	
Mean	Arithmetic mean
SD	Standard deviation
Median	Median
Sum	Sum of values
Max	Highest single value
Min	Lowest single value
Range	Max-Min
3 highest (Sum)	Sum of the 3 highest values
6 highest (Sum)	Sum of the 6 highest values
3 lowest (Sum)	Sum of the 3 lowest values
6 lowest (Sum)	Sum of the 6 lowest values
Sum Day	Sum of values from 04:01 to 20:00
Sum Night	Sum of values from 20:01 to 04:00
Day/Night ratio	Sum Day/Sum Night
Multiple days	
d-1	Value of previous day
d-2	Value 2 days before
d-3	Value 3 days before
RM	Rolling Mean of previous 7 days
RMdiff	Difference of current day’s value to RM
RMprev	Rolling mean of previous week (d-8 to d-14)
slope	Slope of a linear regression from the recent 7 values

**Table 2:** Number of features by category, before feature selection.

Category	Number of Features
Animal dependent variables	
Feed and water intake and visits	189
Activity	127
Milking	77
Concentrate intake	25
Body weight	8
Other ^1^	3
Animal independent variables	
Climate	4

^1^ parity, days and weeks in milk.

**Table 3:** The 20 most important variables ranked by RF-I (mean ± 95% CI) with respective r-values (mean ± CI, all correlations with *p* < 0.001).

	Mastitis Treatments Classification			Lameness Treatments Classification		
Rank	Feature	RF-I ^1^	r	Feature	RF-I	r
1	Last Milk recording SCC ^2^	0.039 ± 0.009	+0.176 ± 0.016	Feeding time with intake	0.013 ± 0.005	−0.105 ± 0.014
2	Concentrate intake, slope	0.014 ± 0.005	−0.076 ± 0.016	Feed intake Sum day, RMprev ^3^	0.012 ± 0.006	+0.079 ± 0.010
3	Milk conductivity p.m., slope	0.013 ± 0.006	+0.082 ± 0.024	Activity, SD, RMprev	0.012 ± 0.006	−0.072 ± 0.012
4	Feed intake (Median), RMprev	0.011 ± 0.004	+0.067 ± 0.010	Feeding visits with intake	0.011 ± 0.005	−0.080 ± 0.013
5	Feed intake (S.D.), RMprev	0.011 ± 0.004	+0.064 ± 0.008	Activity (Range), RMprev	0.010 ± 0.006	−0.067 ± 0.011
6	Feeding visit duration (mean), RM ^4^	0.011 ± 0.004	+0.009 ± 0.006	Activity (Max), RMprev	0.010 ± 0.004	−0.066 ± 0.011
7	Feed intake 6 highest (Sum), RMprev	0.011 ± 0.006	+0.068 ± 0.009	Air temperature	0.010 ± 0.004	−0.061 ± 0.015
8	Feed intake 3 highest (Sum), RMprev	0.010 ± 0.005	+0.068 ± 0.009	THI ^5^	0.009 ± 0.003	−0.061 ± 0.015
9	Feeding visit duration (mean), d−3	0.010 ± 0.004	−0.002 ± 0.006	Feed intake (SD), RM	0.009 ± 0.004	+0.098 ± 0.011
10	Conc. intake abs. deviation, RM	0.010 ± 0.004	−0.047 ± 0.014	Feeding time with intake, RM	0.009 ± 0.006	−0.062 ± 0.008
11	Milk conductivity p.m., RMdiff ^6^	0.010 ± 0.005	+0.080 ± 0.017	Feeding time with intake, RMdiff	0.009 ± 0.003	−0.095 ± 0.018
12	Feed intake (Max), RMprev	0.009 ± 0.006	+0.065 ± 0.01	Drinking time with intake	0.009 ± 0.005	−0.065 ± 0.009
13	Feed intake (Mean), RMprev	0.009 ± 0.005	+0.067 ± 0.01	Feeding time with intake, slope	0.008 ± 0.003	−0.090 ± 0.019
14	Feeding visits with intake, RMprev	0.009 ± 0.006	−0.060 ± 0.005	Feed intake (Median)	0.007 ± 0.001	+0.107 ± 0.010
15	Feed intake (S.D.), RM	0.008 ± 0.003	+0.052 ± 0.007	Feed intake, 6 highest (Sum), RM	0.006 ± 0.003	+0.101 ± 0.009
16	Conc. intake rel. deviation, RM	0.008 ± 0.004	−0.036 ± 0.011	Feed intake per visit	0.006 ± 0.003	+0.106 ± 0.011
17	Feeding visit duration (Mean), RMprev	0.008 ± 0.005	+0.034 ± 0.008	Activity, 3 highest (Sum), RMprev	0.006 ± 0.003	−0.067 ± 0.011
18	Feed intake 6 highest (Sum), RM	0.008 ± 0.003	+0.062 ± 0.008	Feeding visits with intake, d-1	0.006 ± 0.003	−0.068 ± 0.011
19	Feed intake (Range), RMprev	0.008 ± 0.005	+0.065 ± 0.010	Feed intake, RMprev	0.006 ± 0.004	+0.063 ± 0.015
20	Feeding visits with intake, RM	0.008 ± 0.005	−0.057 ± 0.005	Drinking visits, RM	0.006 ± 0.003	−0.059 ± 0.014

^1^ Random Forest-Importance, ^2^ somatic cell count, ^3^ rolling mean of previous week, ^4^ rolling mean, ^5^ temperature humidity index, ^6^ Difference of current day’s value to RM.

**Table 4:** Mean AUC, Sensitivity and Specificity (± 95%-CI) for validation data (33% of sampled training data), means for all machine learning methods.

Sampling of Training Data	AUC ^1^	Sen. ^2^	Spe. ^3^
Mastitis treatments			
No sampling	0.80 ± 0.02 ^b^	0.72 ± 0.04 ^c^	0.72 ± 0.05 ^b^
Random Undersampling	0.76 ± 0.01 ^c^	0.81 ± 0.02 ^b^	0.59 ± 0.04 ^c^
Random Oversampling	0.95 ± 0.01 ^a^	0.89 ± 0.02 ^a^	0.91 ± 0.02 ^a^
SMOTE ^4^	0.95 ± 0.01 ^a^	0.88 ± 0.01 ^a^	0.91 ± 0.02 ^a^
Lameness treatments			
No sampling	0.76 ± 0.02 ^b^	0.70 ± 0.04 ^b^	0.68 ± 0.05 ^b^
Random Undersampling	0.71 ± 0.01 ^c^	0.80 ± 0.02 ^b^	0.53 ± 0.03 ^c^
Random Oversampling	0.91 ± 0.02 ^a^	0.89 ± 0.02 ^a^	0.84 ± 0.04 ^a^
SMOTE	0.91 ± 0.02 ^a^	0.87 ± 0.01 ^a^	0.83 ± 0.04 ^a^

^1^ Area Under ROC-Curve; ^2^ Sensitivity; ^3^ Specificity; ^4^ Synthetic Minority Over-sampling Technique; ^a,b,c^ superscript letters indicate significant differences at *p* ≤ 0.05 between sampling methods within treatments.

**Table 5:** Mean AUC, Sensitivity and Specificity (±95%-CI) for classification of treatments with or without inclusion of data from feed and water troughs, means include all machine learning models and sampling methods.

Feed and Water Data Included	AUC ^1^	Sen. ^2^	Block Sen.	Spe. ^3^
	Mastitis treatments			
Yes	0.67 ± 0.01	0.40 ± 0.02	0.49 ± 0.03	0.82 ± 0.02
No	0.66 ± 0.01	0.39 ± 0.02	0.51 ± 0.02	0.82 ± 0.01
	Lameness treatments			
Yes	0.62 ± 0.01 ^a^	0.41 ± 0.02	0.53 ± 0.02 ^a^	0.76 ± 0.02 ^a^
No	0.55 ± 0.01 ^b^	0.38 ± 0.02	0.50 ± 0.02 ^b^	0.69 ± 0.02 ^b^

^1^ Area Under ROC-Curve; ^2^ Sensitivity; ^3^ Specificity; ^a,b^ superscript letters indicate significant differences at *p* ≤ 0.05 within treatments.

**Table 6:** Mean testing data AUC, Sensitivity, Block Sensitivity and Specificity (± 95%-CI) for each classification model, averaged over all sampling methods, for mastitis and lameness treatments.

Mastitis Treatments		Lameness Treatments	
Classification Method	AUC ^1^	Classification Method	AUC ^1^
LR ^2^	0.75 ± 0.02 ^a^	GNB	0.70 ± 0.01 ^a^
ET ^3^	0.75 ± 0.02 ^a^	Soft Voting 1	0.69 ± 0.01 ^a^
GNB ^4^	0.75 ± 0.02 ^a^	ET	0.68 ± 0.01 ^ab^
Soft Voting 1	0.74 ± 0.02 ^a^	LR	0.68 ± 0.01 ^ab^
Soft Voting 2	0.73 ± 0.02 ^a^	RF	0.67 ± 0.02 ^ab^
RF ^5^	0.72 ± 0.02 ^ab^	Soft Voting 2	0.66 ± 0.02 ^abc^
Grid Search DT ^6^	0.69 ± 0.03 ^bc^	SVM	0.62 ± 0.03 ^bc^
SVM ^7^	0.65 ± 0.03 ^c^	Grid Search DT	0.60 ± 0.02 ^cd^
KNN ^8^	0.58 ± 0.02 ^d^	KNN	0.57 ± 0.02 ^de^
Grid Search ADA ^9^	0.56 ± 0.01 ^d^	Grid Search ADA	0.54 ± 0.01 ^e^
ADA	0.56 ± 0.01 ^d^	ADA	0.54 ± 0.01 ^e^
DT	0.55 ± 0.02 ^d^	DT	0.54 ± 0.01 ^e^

^1^ Area Under ROC-Curve; ^2^ Logistic Regression; ^3^ Extra Trees Classifier; ^4^ Gaussian Naïve Bayes; ^5^ Random Forest; ^6^ Decision Tree; ^7^ Support Vector Machine; ^8^ K-nearest Neighbors; ^9^ AdaBoost; ^a, b, c, d, e^ superscript letters indicate significant (*p* < 0.05) differences between classification methods within treatments.
